# *Cordyceps cicadae* ameliorates inflammatory responses, oxidative stress, and fibrosis by targeting the PI3K/mTOR‐mediated autophagy pathway in the renal of MRL/lpr mice

**DOI:** 10.1002/iid3.1168

**Published:** 2024-01-24

**Authors:** Feng Yang, Yanyan Zhang, Lei Dong, Zhichao Song

**Affiliations:** ^1^ Department of Rheumatology Yantai Hospital of Traditional Chinese Medicine Yantai City Shandong China

**Keywords:** autophagy, *Cordyceps cicadae*, lupus nephritis, PI3K/mTOR, renal fibrosis

## Abstract

**Background:**

The vast majority of systemic lupus erythematosus patients develop lupus nephritis (LN) with severe renal manifestations, such as inflammatory responses, oxidative stress, and fibrosis. The purpose of this research was to investigate *Cordyceps cicadae* as a potential therapeutic target for treating inflammatory responses, oxidative stress, and fibrosis in LN.

**Methods:**

The effects of *C. cicadae* on lupus symptoms in mice with LN were determined. MRL/lpr mice were treated with *C. cicadae* (4 g/kg/day, i.e., CC group, *n* = 8) or an equal volume of saline (model group, *n* = 8), and MRL/MP mice were treated with an equal volume of saline (control group, *n* = 8). Renal function indices, renal pathology, inflammatory markers, oxidative stress markers, and renal interstitial fibrosis levels were evaluated after *C. cicadae* treatment. Western blot analysis was performed to investigate the effect of *C. cicadae* on the expression of fibrosis biomarkers and the phosphatidylinositol 3‐kinase (PI3K)/mammalian target of rapamycin (mTOR)‐mediated autophagy pathway in the renal tissues of mice.

**Results:**

*C. cicadae* ameliorated renal lesions, the inflammatory response, and oxidative stress damage in MRL/lpr mice. *C. cicadae* treatment inhibited renal fibrosis (16.31 ± 4.16 vs. 31.25 ± 5.61) and downregulated the expression of the fibrosis biomarkers alpha‐smooth muscle actin, fibronectin, and collagen I (COL I) in the kidneys of MRL/lpr mice. In addition, further research showed that the PI3K/mTOR‐mediated autophagy pathway was involved in *C. cicadae*‐mediated effects on renal fibrosis in MRL/lpr mice. Furthermore, the therapeutic effect of *C. cicadae* on repairing renal fibrosis and damage in MRL/lpr mice was abolished by the PI3K agonist 740 Y‐P.

**Conclusions:**

The findings of the present research showed that *C. cicadae* could alleviate inflammatory responses, oxidative stress, and fibrosis in the renal tissues of mice with LN by targeting the PI3K/mTOR‐mediated autophagy pathway.

## Graphical abstract

The purpose of this study was to explore the possibility of using *Cordyceps cicadae* as a potential therapeutic target for treating renal fibrosis damage in lupus nephritis. The current study's findings show that *C. cicadae* can alleviate renal fibrosis damage in lupus mice by targeting the phosphatidylinositol 3‐kinase (PI3K)/mammalian target of rapamycin (mTOR)‐mediated autophagy pathway.

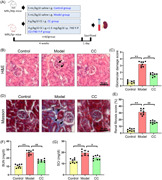

## 1 INTRODUCTION

Systemic lupus erythematosus (SLE) is a prevalent autoimmune illness that can cause damage to a variety of organs and tissues, including the kidneys. Lupus nephritis (LN) is one of the most prevalent lupus complications and affects approximately 80% of children with SLE and 40% of adults with SLE.^1^, ^2^, ^3^ Renal fibrosis in LN patients is the final stage of sustained immune‐mediated injury and is considered a determining factor for treatment response and renal prognosis.^4^ Recent evidence suggests that autophagy is crucial for the pathophysiology of SLE‐induced renal lesions, including fibrosis.^5^ The regulatory mechanism of renal autophagy in LN patients and new treatment strategies targeting renal autophagy still need to be explored.

Renal fibrosis has been identified and researched in a variety of renal illnesses, and it is defined primarily by an imbalance in the breakdown by matrix metalloproteinases and the formation of extracellular matrix (ECM) by myofibroblasts.^4^ Fibrosis is the final common pathway for progressive renal function loss and the development of chronic kidney disease.^6^ During LN, as in other renal disorders, fibrosis is the final result of prolonged immune‐mediated injury and is an important factor in defining responsiveness to therapy and renal prognosis.^7^ Therefore, understanding the pathogenesis of renal fibrogenesis, its contributing mechanisms, potential therapeutic targets, and relevant methodologies for the diagnosis and treatment of renal fibrosis is essential.

Autophagy, which is an important stress‐responsive system, has been implicated in the pathophysiology of several renal disorders, including renal fibrosis.^8^, ^9^ Although autophagy is not required for renal formation, it is important in adult renal resident cells and is closely linked to the progression of renal fibrosis.^10^ Cellular stressors, including hypoxia, reactive oxygen species, stress on the endoplasmic reticulum (ER), damage to DNA, invasive infections, and immunological signals, can trigger autophagy.^8^, ^11^ Mammalian target of rapamycin (mTOR), which is a serine/threonine kinase, acts as a key regulator of metabolic processes in cells and plays an important role in autophagy regulation.^12^ As a downstream target of the phosphatidylinositol 3‐kinase (PI3K)/Akt pathway, mTOR acts as a negative regulator of autophagy by inhibiting the generation of unc‐51‐like autophagy‐activating kinase (ULK) complexes and blocking the formation of autophagosomes.^13^, ^14^ Therefore, targeting the PI3K/mTOR pathway could be a promising approach for treating tissue fibrosis by regulating autophagy. Although studies have shown that *Cordyceps cicadae* and its extracts can affect the PI3K/mTOR pathway and autophagy activity in renal tissue, the effects of these extracts on lupus‐induced renal fibrosis has not been determined.

*C. cicadae* is a traditional Chinese medicine (TCM) that parasitizes *Cicada flammata* larvae and is a member of the Cordycipitaceae family.^15^ Pharmacological research has revealed that the fungus contains a number of biologically active chemical compounds, including myriocin, cordycepic acid, cordycepin, beauvericin, and nucleosides.^16^ Historically, blood fat reduction, analgesia‐antipyresis, liver and kidney protections, and anticancer activities have all been attributed to *C. cicadae*.^17^, ^18^ Recently, research has shown that *C. cicadae* can ameliorate hypertensive renal damage by reducing renal fibrosis.^17^, ^19^ In addition, in vivo studies have revealed that treatment with *C. cicadae* can reduce the degree of renal interstitial fibrosis in mice with unilateral ureteral obstruction.^18^ However, it is still unknown whether *C. cicadae* protects against SLE‐induced renal injury, especially fibrosis.

In this research, we aimed to investigate whether *C. cicadae* could relieve renal damage, especially inflammation, oxidative stress, and fibrosis, in mice with lupus. By exploring its regulatory effects on the PI3K/mTOR‐mediated autophagy pathway, we hoped to reveal the mechanism by which *C. cicadae* can repair renal fibrosis in mice with lupus and provide new strategies for clinical treatments.

## 2 MATERIALS AND METHODS

### 2.1 Animals and groups

As previously reported, MRL/Mp‐lpr/lpr (MRL/lpr) mice could be developed into systemic autoimmune illnesses such as lymph node enlargement, aberrant T cell proliferation, arthritis, and immune complex type glomerulonephritis were used as a lupus animal model, MRL/Mp‐+/+ (MRL/Mp) mice were used as the control for MRL/lpr.^20^ MRL/lpr and MRL/Mp mice (female and 6−8 weeks old) were obtained from Shanghai Laboratory Animals Center (SLAC) Laboratory Animal Co., Ltd. and housed in a specific pathogen‐free laboratory with standard temperature (25°C) and humidity (40%−60%), as well as a 12 h light/dark cycle and standard pallet diet and water.

*C. cicadae* was obtained from the Zhejiang BioAsia Pharmaceutical Co., Ltd. *C. cicadae* was dissolved in saline and adjusted to a final concentration of 10 mg/mL as previously reported.^19^ MRL/lpr mice were randomly divided into two groups: mice treated with *C. cicadae* (4 g/kg/day, i.e., CC group, *n* = 8) and mice treated with an equal volume of saline (model group, *n* = 8) for 4 weeks and killed 1 day after the last treatment. And MRL/MP mice were also treated with an equal volume of saline (control group, *n* = 8). To verify whether the anti‐fibrotic effect of *C. cicadae* depends on the PI3K/mTOR‐mediated autophagy pathway, we conducted PI3K agonist (740 Y‐P, cat. no. 1236188‐16‐1; Aladdin) intervention on the CC group. *C. cicadae* treated MRL/lpr mice were intraperitoneally injected with 740 Y‐P (1.5 mg/kg/day) or equivalent amounts of dimethyl sulfoxide (cat. no. 67‐68‐5; Aladdin) alone as a control.

After that, the kidneys were divided, with the right kidney used for western blot analysis and the left kidney used for pathological analysis. Serum and kidney samples were stored and kept at −80°C for later analysis. All experimental procedures were approved by the Yantai Hospital of Traditional Chinese Medicine Ethics Board (approval no. 2022‐07).

### 2.2 Assessment of renal functions

After the mice were killed, blood samples were taken from the abdominal aorta. To separate the serum, the blood was centrifuged at 3000*g* for 15 min at 4°C. Serum creatinine (SCr) and blood urea nitrogen (BUN) concentrations were measured using a SCr (cat. no. #C011‐2‐1) kit and a BUN (cat. no. #C013‐2‐1) kit obtained from Nanjing Jiancheng. The manufacturer's instruction for the corresponding assay kits were followed.

### 2.3 Histological examination

Hematoxylin and eosin (H&E) and Masson's trichrome staining were performed as described previously.^21^, ^22^ Kidney tissues were fixed using 10% formaldehyde for 24 h at 4°C, embedded in paraffin, cut into 4 µm sections and mounted on slides. The prepared slides were deparaffinized twice in xylene at room temperature and rehydrated using an ethanol gradient before being stained independently with Masson's trichrome (5 min, room temperature) and H&E (5 min, room temperature). A microscope was used to obtain the images (Leica DM4000). ImageJ (NIH) was used to calculate fibrosis as the percentage of blue collagen‐stained area relative to total tissue in one field. All samples were assessed by two independent investigators in a blinded manner.

### 2.4 Detection of serum cytokines

The serum inflammatory cytokines including monocyte chemoattractant protein‐1 (MCP‐1, cat. no. SEKP‐0019), tumor necrosis factor‐alpha (TNF‐α, cat. no. SEKM‐0034), interferon‐gamma (IFN‐γ, cat. no. SEKM‐0031), and interleukin‐6 (IL‐6, cat. no. SEKM‐0007) were measured using the Multiskan FC Microplate Reader (Thermo Fisher Scientific). All enzyme‐linked immunosorbent assay (ELISA) kits were obtained from Solarbio and manufacturer's protocols were performed.

### 2.5 Detection of oxidative stress

The oxidative stress levels in renal tissue were evaluated by detecting 8‐hydroxy‐2'‐deoxyguanosine (8‐OH‐dG) and malondialdehyde (MDA) levels. ELISA kit was used to detect 8‐OH‐dG (cat. no. STA‐320‐T; Cell BioLabs) levels in renal tissue. The colorimetric method was used to detect MDA (cat. no. S0131S; Beyotime) levels in renal tissue. The manufacturer's instruction for the corresponding assay kits were followed.

### 2.6 Detection of antioxidant capacity

The superoxide dismutase (SOD) (cat. no. A001‐3‐2) and catalase (CAT) (cat. no. A007‐1‐1) activities of renal tissues were measured by the Orion AquaMate UV‐Vis spectrophotometer with commercial kit (all from Nanjing Jiancheng). The manufacturer's instruction for the corresponding assay kits were followed.

### 2.7 Western blot analysis

Western blot assays were carried out as previously described.^23^ Following sodium dodecyl sulfate‐polyacrylamide gel electrophoresis technique, proteins were electro‐transferred to polyvinylidene fluoride membranes and blocked for 2 h at room temperature in 5% BSA dissolved in Tris‐buffered saline with Tween‐20 (TBST). The membranes were then treated overnight at 4°C with primary antibodies against alpha‐smooth muscle actin (α‐SMA) (1:1000 dilution; #19245), fibronectin (FN) (1:1000 dilution; #26836), and collagen I (COL I) (1:1000 dilution; #72026), LC3‐II/I (1:1000 dilution; #12741), P62 (1:1000 dilution; #23214), Beclin‐1 (1:1000 dilution; #4122), p‐mTOR (1:1000 dilution; #5536), mTOR (1:1000 dilution; #2983), p‐PI3K (1:1000 dilution; #17366), PI3K (1:1000 dilution; # 4257), and β‐actin (1:1000 dilution; #4970) all acquired from CST Corporation. The membranes were then incubated with a secondary antibody (anti‐rabbit IgG [H + L]; #14708; CST) for 2 h at room temperature, followed by TBST washes. Signal detection was performed using an Electrochemiluminescence kit by the ChemiDoc Touch imaging system (Bio‐Rad Laboratories).

### 2.8 RNA extraction and quantitative real‐time PCR

A quantitative real‐time PCR test was used to detect gene expression as previously described.^24^ The total RNA was extracted using the TRIzol kit (Invitrogen). RT‐qPCR was carried out using the SYBR® Premix Ex TaqTM kit (Takara Bio) according to the manufacturer's instructions. The qPCR reaction conditions were subjected to an initial predenaturation step at 95°C for 3 min, followed by 39 cycles of 95°C for 20 s and 60°C for 30 s. Using glyceraldehyde‐3‐phosphate dehydrogenase (GAPDH) as internal control, we calculated the relative abundance of genes using $2^{-∆∆C_{t}}$ formula. The primer sequences were as follows: IL‐6 forward, 5′‐GGTTTGCCGAGTAGACCTCA‐3′ and reverse, 5′‐GTGGCTAAGGACCAAGACCA‐3′; IFN‐γ forward, 5′‐CTTCTTCAGCAACAGCAAGG‐3′ and reverse, 5′‐TGAGCTCATTGAATGCTTGG‐3′; MCP‐1 forward, 5′‐TTAAAAACCTGGATCGGAACCAA‐3′ and reverse, 5′‐GCATTAGCTTCAGATTTACGGGT‐3′; TNF‐α forward, 5′‐CCCGGAATGTCGATGCCTGAGTG‐3′ and reverse, 5′‐CGCCCCGGCCTTCCAAATAAAT‐3′; GAPDH forward, 5′‐TGGCCTTCCGTGTTCCTAC‐3′ and reverse, 5′‐GAGTTGCTGTTGAAGTCGCA‐3′.

### 2.9 Statistical analysis

Statistical analyses were carried out using Statistical Product and Service Solutions (SPSS) (19.0 Inc.). Three independent experiments are represented as means ± standard deviation. The data were evaluated using the Independent Student's *t*‐test or one‐way ANOVA and Tukey's post hoc test.^25^ Significance was established when *p* < .05.

## 3 RESULTS

### 3.1 *C. cicadae* alleviates renal lesions in MRL/lpr mice

**Figure 1:**
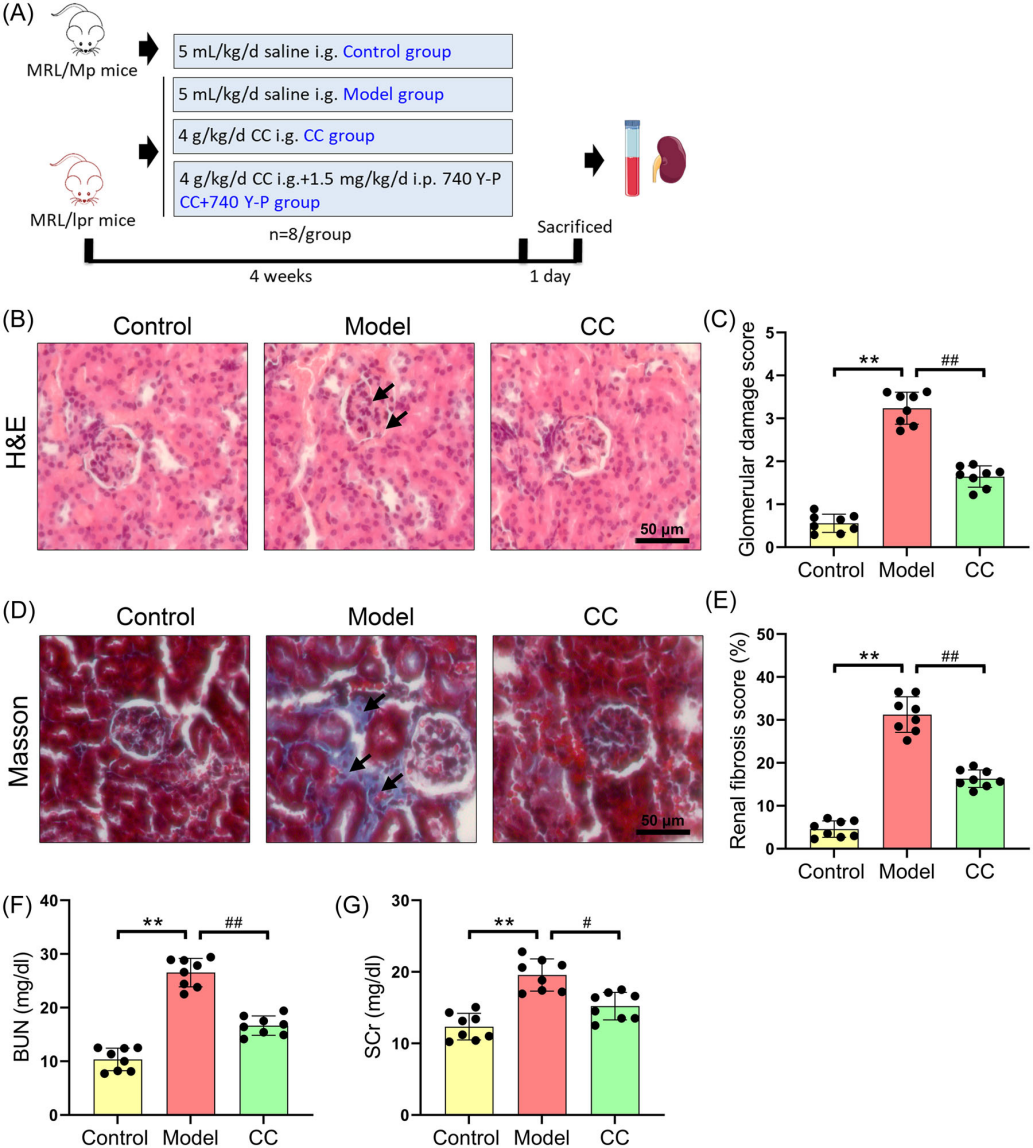
*Cordyceps cicadae* alleviates renal lesions in MRL/lpr mice. (A) The scheme of the experimental design. (B) Representative photomicrographs of H&E stained renal sections (bar = 50 μm). (C) Quantification of glomerular injury score. (D) Representative photomicrographs of Masson's trichrome‐stained renal sections (bar = 50 μm). (E) Quantification of renal fibrosis score. Renal functions were assessed by BUN (F) and SCr (G) levels. All data are expressed as the mean ± SD. ^∗∗^ *p* < .01, control group versus model group; ^#^ *p* < .05 and ^##^ *p* < .01, model group versus CC group. BUN, blood urea nitrogen; H&E, hematoxylin and eosin.

Figure 1A shows a schematic diagram of the animal research to confirm the effectiveness of *C. cicadae* in treating renal damage in mice with LN. H&E staining showed ECM deposition and glomerular swelling, as well as a large amount of inflammatory cell infiltration in the model group compared to the control group, as shown in Figure 1B. Therefore, the glomerular damage score was significantly higher in the model group than in the control group (Figure 1C). Furthermore, Masson staining demonstrated that the model group had a considerably larger renal interstitial fibrosis area than the control group (Figure 1D,E). *C. cicadae* treatment, on the other hand, dramatically reduced the abovementioned glomerular damage and renal interstitial fibrosis in MRL/lpr mice (Figure 1B−E). The concentrations of BUN and SCr were measured to assess the effect of *C. cicadae* on renal function parameters in mice with LN. As shown in Figure 1F,G, the BUN and SCr levels in the model group were significantly higher than those in the control group, but *C. cicadae* treatment dramatically decreased the BUN and SCr levels in MRL/lpr mice.

### 3.2 *C. cicadae* alleviates the renal inflammatory response and oxidative stress in MRL/lpr mice

**Figure 2:**
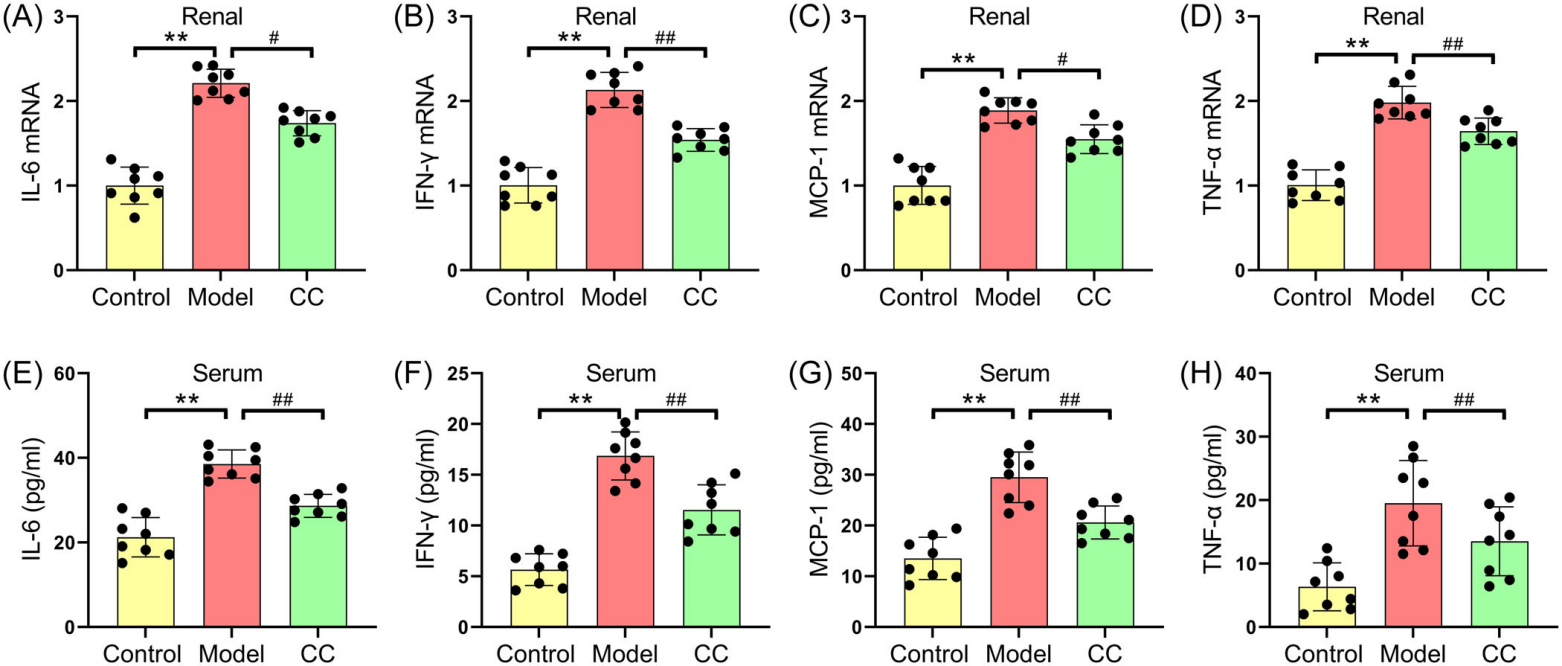
*Cordyceps cicadae* inhibits the expressions of inflammatory factors in serum and renal tissue of MRL/lpr mice. The relative mRNA levels of IL‐6 (A), IFN‐γ (B), MCP‐1 (C), and TNF‐α (D) in renal tissues of mice. The concentrations of IL‐6 (E), IFN‐γ (F), MCP‐1 (G), and TNF‐α (H) in the serum of mice. All data are expressed as the mean ± SD. ^∗∗^ *p* < .01, control group versus model group; ^#^ *p* < .05 and ^##^ *p* < .01, model group versus CC group. IFN‐γ, interferon‐gamma; IL‐6, interleukin‐6; MCP‐1, monocyte chemoattractant protein‐1; TNF‐α, tumor necrosis factor‐alpha.

**Figure 3:**
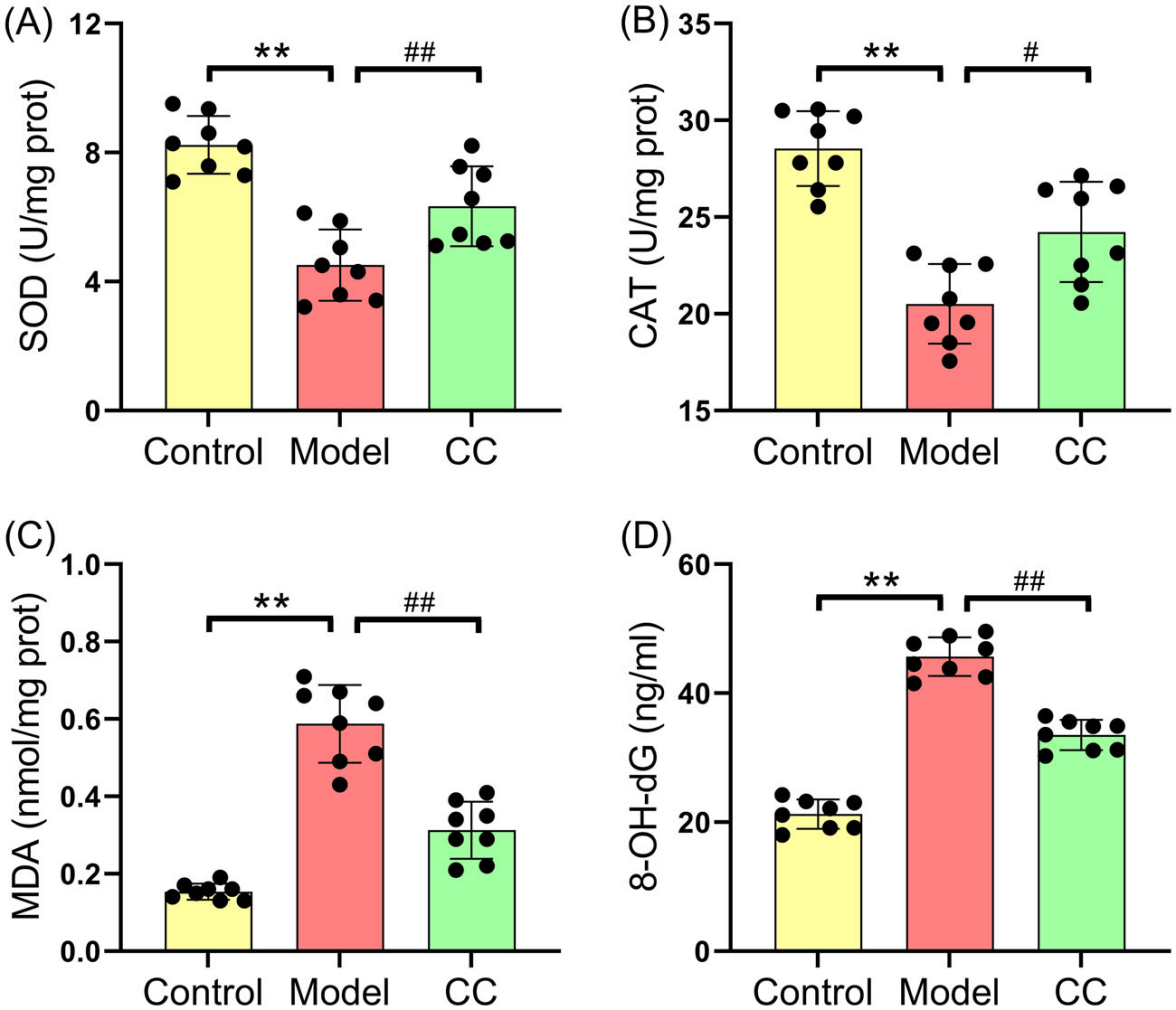
*Cordyceps cicadae* repairs redox metabolic imbalance in the renal of MRL/lpr mice. The activities of antioxidant enzymes SOD (A) and CAT (B) in renal tissues of mice. The concentrations of oxidative stress parameters MDA (C) and 8‐OH‐dG (D) in renal tissues of mice. All data are expressed as the mean ± SD. ^∗∗^ *p* < .01, control group versus model group; ^#^ *p* < .05 and ^##^ *p* < .01, model group versus CC group. CAT, catalase; MDA, malondialdehyde; SOD, superoxide dismutase.

The effects of *C. cicadae* on the inflammatory response and redox metabolism in MRL/lpr mice were investigated. As shown in Figure 2, the levels of IL‐6, IFN‐γ, MCP‐1, and TNF‐α in the serum and renal tissues of the model group were significantly higher than those in the control group. In addition, *C. cicadae* administration dramatically reduced the serum and renal tissue levels of the inflammatory markers IL‐6 (38.55 ± 3.33 vs. 28.65 ± 2.73 pg/mL for serum cytokines and 2.21 ± 0.17 vs. 1.74 ± 0.15 for renal tissue mRNA), IFN‐γ (16.86 ± 2.36 vs. 11.54 ± 2.47 pg/mL for serum cytokines and 2.13 ± 0.21 vs. 1.54 ± 0.13 for renal tissue mRNA), MCP‐1 (29.51 ± 4.99 vs. 20.61 ± 3.25 pg/mL for serum cytokines and 1.89 ± 0.15 vs. 1.55 ± 0.17 for renal tissue mRNA), and TNF‐α (19.51 ± 6.74 vs. 13.51 ± 4.44 pg/mL for serum cytokines and 1.98 ± 0.19 vs. 1.64 ± 0.15 for renal tissue mRNA) in MRL/lpr mice. Furthermore, compared with those in the control group, the activities of SOD and CAT in the model group markedly decreased, and the levels of MDA and 8‐OH‐dG were markedly increased (Figure 3). Conversely, *C. cicadae* treatment markedly increased the activities of SOD (4.51 ± 1.11 vs. 6.33 ± 1.24 U/mg prot) and CAT (20.51 ± 2.06 vs. 24.23 ± 2.56 U/mg prot) and decreased the levels of MDA (0.59 ± 0.10 vs. 0.31 ± 0.07 nmol/mg prot) and 8‐OH‐dG (45.68 ± 3.00 vs. 33.51 ± 2.34 ng/mL) in the renal tissues of MRL/lpr mice (Figure 3).

### 3.3 *C. cicadae* attenuates renal fibrosis in MRL/lpr mice

**Figure 4:**
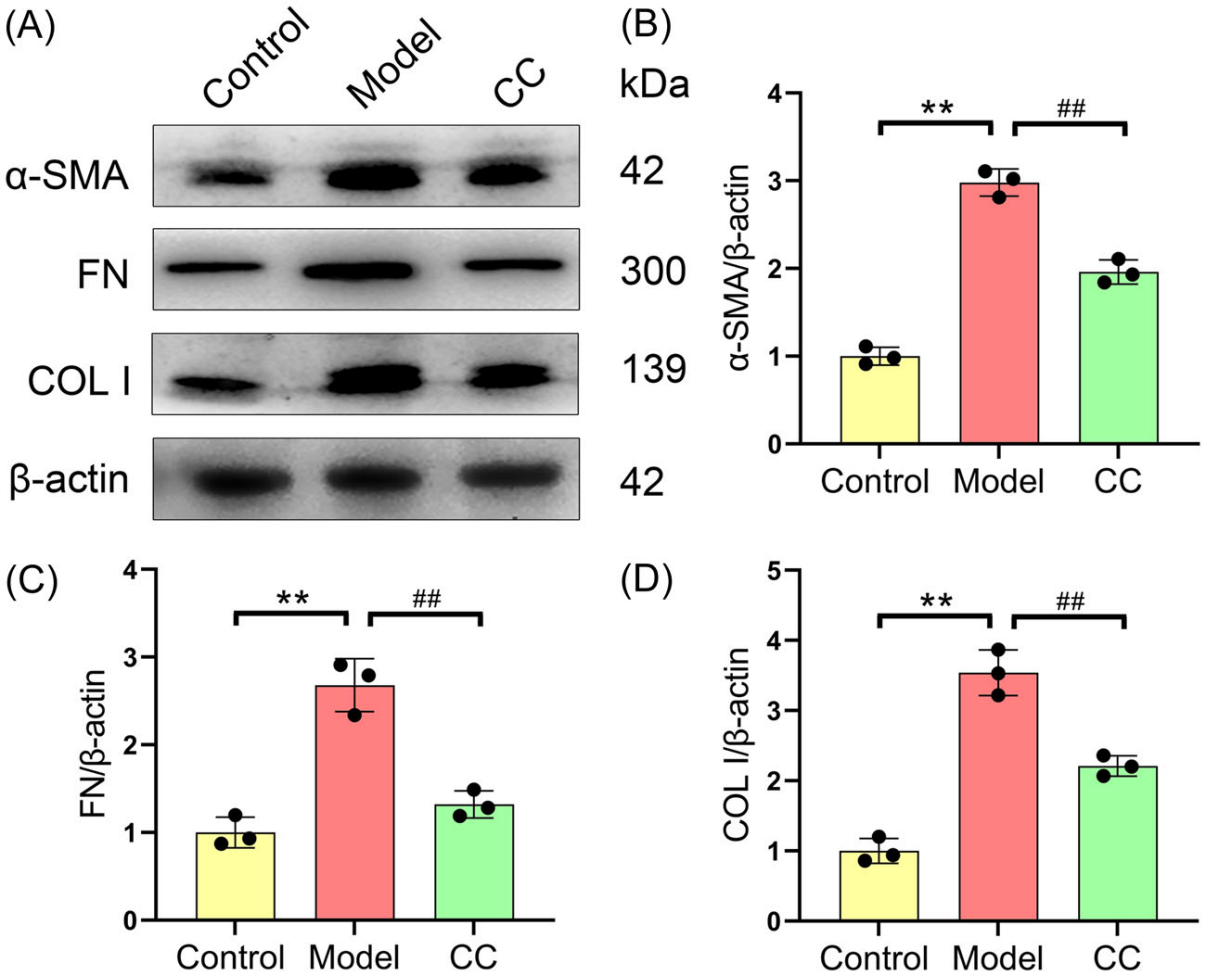
*Cordyceps cicadae* attenuates renal fibrosis in MRL/lpr mice. Representative western blot analysis images (A) and summarized data for the expression of α‐SMA (B), FN (C), and COL I (D) in the renal tissues of mice. All data are expressed as the mean ± SD. ^∗∗^ *p* < .01, control group versus model group; ^##^ *p* < .01, model group versus CC group. COL I, collagen I; FN, fibronectin; α‐SMA, alpha‐smooth muscle actin.

We assessed the impact of *C. cicadae* on the expression of fibrosis biomarkers in the renal tissue of mice with LN. As shown in Figure 4, compared to that in the control group, renal tissue expression of α‐SMA, FN, and COL I in the model group was greatly increased. Conversely, *C. cicadae* treatment significantly suppressed the expression of α‐SMA (2.98 ± 0.15 vs. 1.96 ± 0.14), FN (2.68 ± 0.30 vs. 1.32 ± 0.15), and COL I (3.54 ± 0.33 vs. 2.21 ± 0.15) in the renal tissues of MRL/lpr mice.

### 3.4 *C. cicadae* enhances autophagy by inhibiting of the PI3K/mTOR pathway

**Figure 5:**
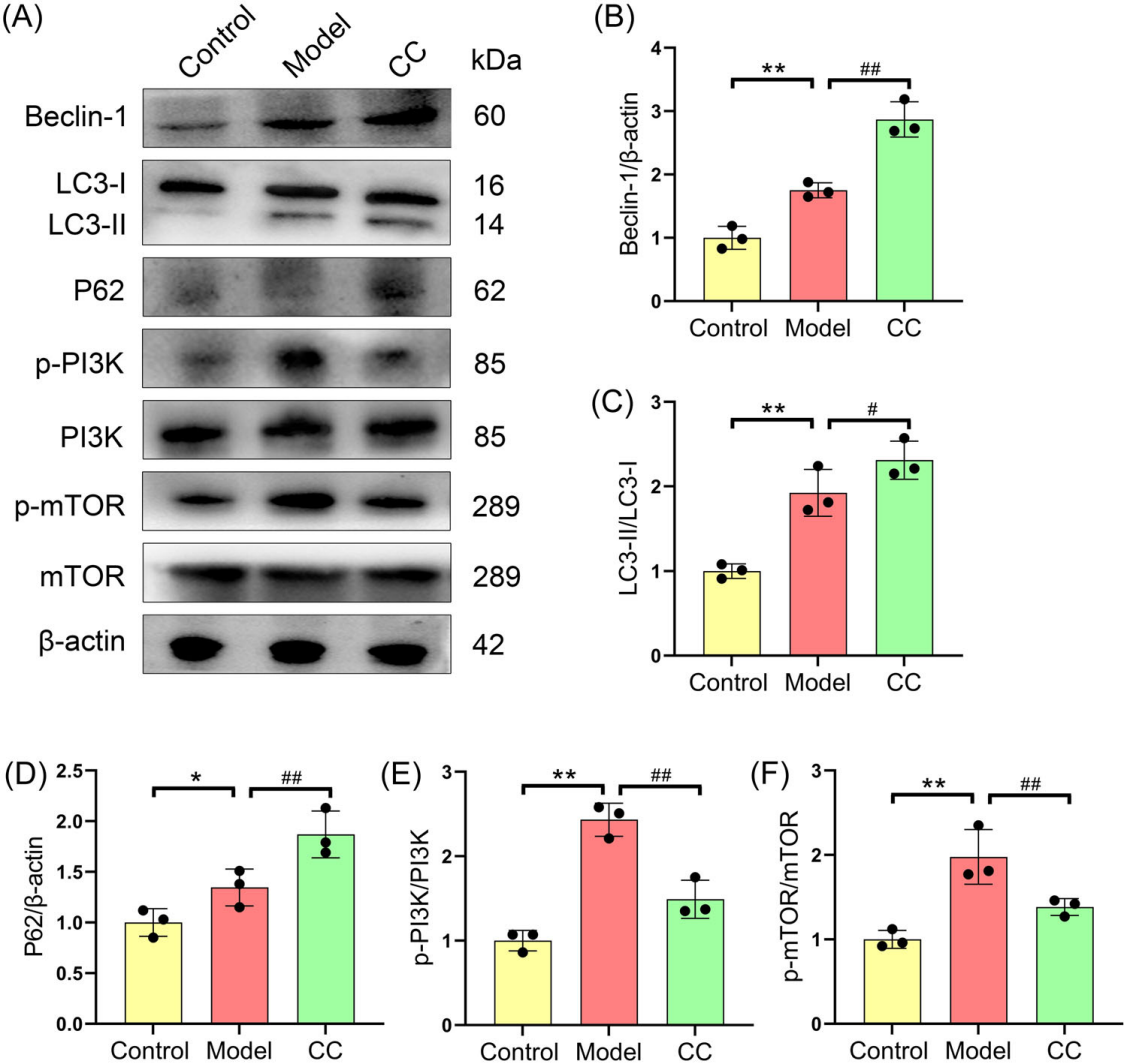
*Cordyceps cicadae* enhances autophagy by targeting the PI3K/mTOR pathway in the renal of MRL/lpr mice. Representative western blot analysis images (A). The expressions of LC3‐II/I (B), P62 (C), and Beclin‐1 (D) and the ratio of p‐PI3K/PI3K (E) and p‐mTOR/mTOR (F) in the renal tissues of mice. All data are expressed as the mean ± SD. ^∗∗^ *p* < .05 and ^∗∗^ *p* < .01, control group versus model group; ^#^ *p* < .05 and ^##^ *p* < .01, model group versus CC group. mTOR, mammalian target of rapamycin; PI3K, phosphatidylinositol 3‐kinase.

To investigate the mechanism by which *C. cicadae* alleviates renal fibrosis in mice with lupus, we examined the expression of factors in the PI3K/mTOR‐mediated autophagy pathway. As shown in Figure 5A−D, there was a significant increase in the expression of the autophagy‐related molecules LC3‐II/I, P62, and Beclin‐1 in the renal tissues of the model group compared to those in the control group. Moreover, the p‐mTOR/mTOR and p‐PI3K/PI3K ratios were significantly higher in the renal tissue of the model group than in that of the control group (Figure 5E,F). Furthermore, we found that *C. cicadae* treatment significantly inhibited the p‐mTOR/mTOR and p‐PI3K/PI3K ratios while significantly increasing the expression of the autophagy‐related molecules LC3‐II/I, P62, and Beclin‐1 in MRL/lpr mice (Figure 5).

### 3.5 740 Y‐P reverses the inhibitory effect of *C. cicadae* on renal fibrosis

**Figure 6:**
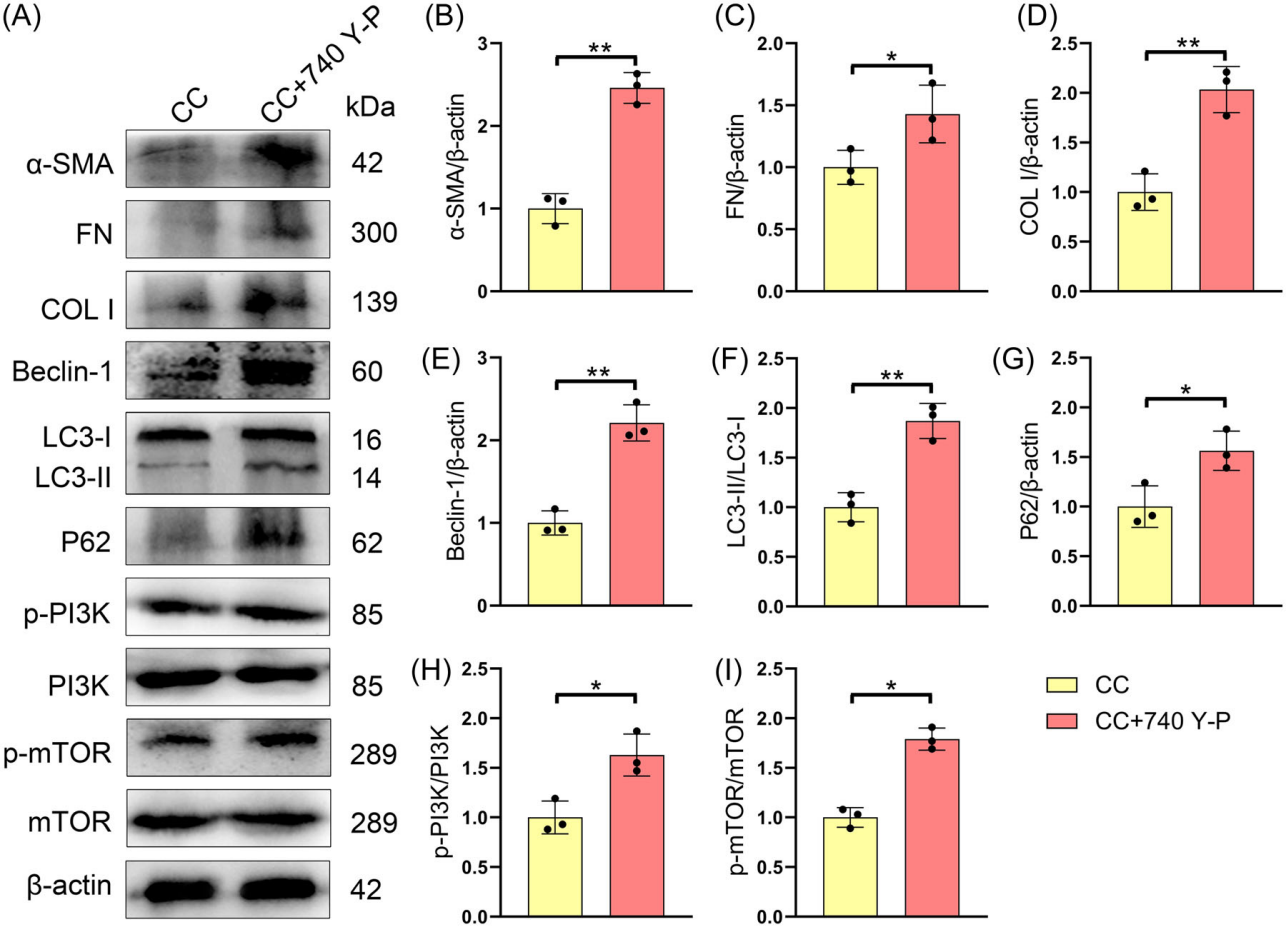
740 Y‐P reverses the inhibitory effect of *Cordyceps cicadae* on renal fibrosis in the renal of MRL/lpr mice. Representative western blot analysis images (A). The expressions of α‐SMA (B), FN (C), and COL I (D) in the renal tissues of mice. The expressions of LC3‐II/I (E), P62 (F), and Beclin‐1 (G) and the ratio of p‐PI3K/PI3K (H) and p‐mTOR/mTOR (I) in the renal tissues of mice. All data are expressed as the mean ± SD. ^∗^ *p* < .05 and ^∗∗^ *p* < .01, CC group versus CC + 740 Y‐P group. COL I, collagen I; FN, fibronectin; mTOR, mammalian target of rapamycin; PI3K, phosphatidylinositol 3‐kinase; α‐SMA, alpha‐smooth muscle actin.

To verify whether the antifibrotic effect of *C. cicadae* depended on the PI3K/mTOR‐mediated autophagy pathway, we treated the CC group with a PI3K agonist (740 Y‐P). As shown in Figure 6A, H‐I and 740 Y‐P treatment markedly increased the p‐mTOR/mTOR and p‐PI3K/PI3K ratios in the CC group. In addition, 740 Y‐P significantly increased the expression of the autophagy‐related molecules LC3‐II/I, P62, and Beclin‐1 in the CC group (Figure 6A,E,−G). The expression of α‐SMA, FN, and COL I in the renal tissues of the CC group also increased dramatically after 740 Y‐P treatment (Figure 6A−D).

## 4 DISCUSSION

Imbalances in immunological homeostasis cause renal inflammatory responses and oxidative stress damage, which are the most common consequences of lupus and have major impacts on prognosis.^26^ Although *C. cicadae* is a TCM used to treat chronic renal problems, its direct targets and biological functions have not been identified.^27^ In this research, we showed that *C. cicadae* treatment could reduce inflammatory responses, redox metabolism, and fibrosis in renal tissues by targeting the PI3K/mTOR‐mediated autophagy pathway in mice with LN. These findings reveal some novel molecular events by which *C. cicadae* protects against lupus‐induced renal impairment.

A growing body of evidence demonstrates that inflammation and oxidative stress play vital roles in the pathophysiology of lupus‐induced renal impairment.^28^, ^29^, ^30^, ^31^ We observed glomerular damage in MRL/lpr mice, which was accompanied by a significant increase in the renal function parameters BUN and SCr. In addition, the levels of inflammatory markers (IL‐6, IFN‐γ, MCP‐1, and TNF‐α) and oxidative stress markers (MDA and 8‐OH‐Dg) were significantly increased in the kidneys of MRL/lpr mice. Recent studies have shown that the use of a water extract of *C. cicadae* (500 mg/kg) may be a promising therapeutic strategy for the prevention of cisplatin‐induced renal damage in mice by inhibiting inflammation and oxidative stress.^27^ Similarly, our present research also showed that treating mice with LN with *C. cicadae* significantly reduced inflammation and oxidative damage and improved renal tissue function and structure. These data showed that reversing oxidative stress and inflammatory reactions are potential mechanisms by which *C. cicadae* alleviates LN. Based on previous literature reports, the noncanonical nuclear factor‐kappa B (NF‐κB) and mitogen‐activated protein kinase (MAPK) signaling pathways may be involved in the regulation of the inflammatory response by *C. cicadae* in mice with kidney injury, and the Nrf2 antioxidant pathway may be involved in the regulation of oxidative stress injury by *C. cicadae* in mice with kidney injury.^27^ Thus, in subsequent investigations, it may be possible to pinpoint the molecular mechanism by which *C. cicadae* affects oxidative damage and the inflammatory response in the renal tissues of mice with LN by targeting relevant signaling molecules such as NF‐κB, MAPK, Nrf2, and HO‐1.

Renal fibrosis has been observed in several kidney diseases, including LN.^4^, ^32^ This research revealed that the area of renal interstitial fibrosis and the expression levels of fibrosis biomarkers (α‐SMA, FN, and COL I) in the renal tissues of mice with LN were significantly higher than those in the control group. However, *C. cicadae* treatment significantly reduced the degree of fibrosis damage in renal tissues from mice with LN. Similarly, Cai et al. reported that *C. cicadae* improved kidney fibrosis in rats with hypertensive nephropathy by inhibiting the expression of α‐SMA, FN, and COL I.^19^ These findings suggest that these molecules may be targets through which *C. cicadae* suppresses kidney fibrosis.

Autophagy has recently been linked to the progression of renal fibrosis, in the tubulointerstitium and glomeruli.^9^, ^33^ However, the precise mechanisms of autophagy in different types of renal cells during renal fibrosis have not been determined. In most organs, autophagy plays dual roles in preventing and exacerbating injury. To maintain the internal environment of the kidney, cells must be stable, viable, and physiologically functional, and autophagic activity is crucial.^34^ Autophagy has been shown to be a vital component in maintaining renal function in experimental models of renal fibrosis^35^; however, other research has demonstrated that autophagy activation can result in tissue damage.^36^, ^37^ In this research, we showed that the expression of autophagy‐associated molecules (LC3‐II/I, P62, and Beclin‐1) and renal fibrosis‐associated molecules (α‐SMA, FN, and COL I) was elevated in the renal tissues of mice with LN compared to control mice. This finding is consistent with prior observations indicating that autophagy is involved in and triggered by LN pathogenesis.^38^ Furthermore, autophagy activation has a negative relationship with podocyte injury, which is responsible for proteinuria and the progression of glomerular disorders.^39^ According to previous studies, autophagy protects resident renal cells, such as podocytes, tubular epithelial cells, glomerular mesangial cells, and endothelial cells, from damage and prevents the development of renal fibrosis.^8^, ^40^, ^41^ Therefore, the activation of renal autophagy in mice with lupus may be a manifestation of self‐protection mechanisms. Interestingly, *C. cicadae* treatment significantly promoted the expression of autophagy pathway factors and significantly alleviated the degree of renal tissue fibrosis in mice with LN. These results suggested that *C. cicadae* may ameliorate renal tissue fibrosis in mice with LN by targeting the autophagy pathway.

According to previous reports, the PI3K/mTOR pathway plays a crucial role in regulating autophagy. Liu et al. reported that curcumin, which targets the mTOR/autophagy axis, could attenuate cardiac hypertrophy and fibrosis in a rat model.^42^ Furthermore, Jia et al. discovered that blocking the PI3K/AKT/mTOR signaling pathway promoted autophagy and reduced peritoneal fibrosis during peritoneal dialysis.^43^ In this research, we found that p‐mTOR and p‐PI3K were markedly upregulated the renal tissues of mice with LN, and *C. cicadae* treatment significantly decreased the levels of activated mTOR in vivo. Similarly, Yang et al. reported that *Paecilomyces cicadidae* could protect podocytes and increase autophagy by blocking the PI3K/AKT/mTOR pathway.^44^ Here, we found that the therapeutic effect of *C. cicadae* on repairing renal fibrosis damage in mice with LN was abolished by the PI3K agonist 740 Y‐P. These results demonstrated that *C. cicadae* was effective at reducing renal fibrosis in mice with lupus by inhibiting the PI3K/mTOR pathway and promoting renal autophagy. In contrast, Cai et al. reported that *C. cicadae* could alleviate renal autophagic stress by regulating the SIRT1 pathway and protecting renal function from fibrosis.^19^ This suggests that *C. cicadae* may have dual roles in the regulation of the autophagic pathway in renal tissues, but the specific mechanism involved remains to be investigated.

This research has two important potential limitations. The mechanism through which autophagy affects various kinds of renal cells during renal fibrosis is unclear. In vitro cellular investigations should be performed to clarify the effects of different oxidative stress and inflammatory conditions on autophagy in various types of renal cells, as well as the regulatory effects and mechanisms of *C. cicadae*. Furthermore, the active components and molecular processes by which *C. cicadae* exerts its therapeutic effects must be clarified.

In conclusion, the present research identified a potential anti‐inflammatory, antioxidant, and antifibrotic role for *C. cicadae* in protecting against lupus‐induced renal impairment through the regulation of the PI3K/mTOR‐mediated autophagy pathway. Our research sheds new light on the therapeutic potential of *C. cicadae* for treating SLE and LN. In the future, the effects of *C. cicadae* on the autophagy pathway in different kidney tissue cells, as well as its therapeutic mechanisms in relation to various kidney diseases, will be further elucidated.

## AUTHOR CONTRIBUTIONS

**Feng Yang**: Conceptualization; formal analysis; project administration. **Yanyan Zhang**: Data curation; formal analysis; software. **Lei Dong**: formal analysis; software. **Zhichao Song**: Conceptualization; data curation; methodology; writing—review and editing.

## CONFLICT OF INTEREST STATEMENT

The authors declare no conflict of interest.

## ETHICS STATEMENT

This study protocol was reviewed and approved by the Ethics Committee of Yantai Hospital of Traditional Chinese Medicine (approval no. 2022‐07).

## Data Availability

The data sets used and/or analyzed during the current study are available from the corresponding author on reasonable request.
